# Engineered nano-bacteria hybrids for precision cancer immunotherapy

**DOI:** 10.1016/j.mtbio.2025.102484

**Published:** 2025-10-31

**Authors:** Liang Yu, Yulin Qiu, Baorui Liu, Xu Zhen, Rutian Li

**Affiliations:** a The Comprehensive Cancer Centre of Nanjing Drum Tower Hospital, The Affiliated Hospital of Nanjing University Medical School and Clinical Cancer Institute of Nanjing University, Nanjing, 210008, China; b MOE Key Laboratory of High Performance Polymer Materials & Technology and State Key Laboratory of Analytical Chemistry for Life Science, School of Chemistry & Chemical Engineering, Nanjing University, Nanjing, 210023, China

## Abstract

Bacterial therapy has emerged as a promising modality in precision cancer immunotherapy, leveraging bacteria's innate tumor-homing ability, immunostimulatory properties, and engineering flexibility to overcome limitations in conventional treatments. This review highlights recent advances in integrating nanotechnology with bacterial systems to develop multifunctional platforms for precision cancer immunotherapy. We first discuss the development of nano-bacteria hybrids, including physical, chemical, and biological interface strategies for constructing multifunctional hybrids, as well as the therapeutic utility of bacterial-derived components such as outer membrane vesicles (OMVs), bacterial ghosts (BGs), and gas vesicles (GVs). We then discuss the synergistic integration of nano-bacteria hybrids with immune checkpoint blockade, CAR-T cell therapy, and genetic engineering approaches to enhance drug delivery, modulate the tumor microenvironment (TME), and improve immune activation. Finally, we explore innovative strategies to overcome current limitations in bacterial immunotherapy, such as tumor heterogeneity, immunosuppression, and safety concerns, while proposing future directions for the clinical translation of nano-bacteria hybrids.

## Graphical abstract

**Figure:**
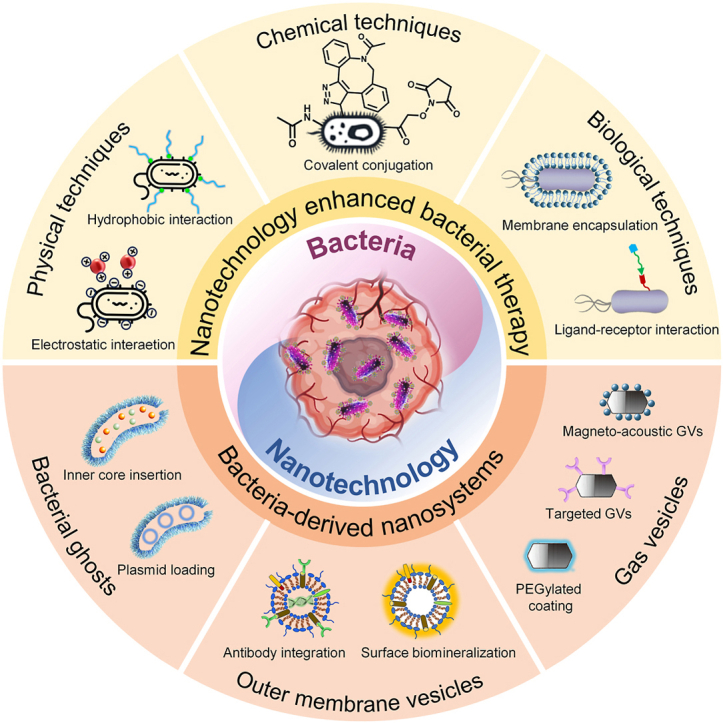

## 1 Introduction

Cancer immunotherapy, which leverages the immune system's inherent capability for precise recognition and elimination of tumor cells, has become an indispensable adjunct to traditional antitumor treatments. Current clinical approaches include cancer vaccines, oncolytic viruses, dendritic cell (DC)-based therapies, adoptive cell transfer, immune checkpoint inhibitors (ICIs), and antibody-drug conjugates (ADCs) [[1], [2], [3]]. Among these strategies, immune checkpoint blockade (ICB), specifically targeting the programmed cell death protein 1/programmed death-ligand 1 (PD-1/PD-L1) axis and cytotoxic T-lymphocyte-associated protein 4 (CTLA-4), stands as a cornerstone of contemporary cancer immunotherapy. This innovative therapeutic modality has markedly transformed cancer treatment paradigms, resulting in substantial clinical benefits for subsets of patients. Nevertheless, a considerable proportion of patients fail to achieve durable responses due to complex resistance mechanisms. Thus, the development of innovative combination therapies designed to circumvent these resistance pathways and improve long-term clinical outcomes remains a pressing clinical need [4].

The potential of bacteria as anticancer agents was recognized as early as the 19th century. William Coley, often regarded as the father of cancer immunotherapy, reported significant tumor regression in patients following injections of heat-killed streptococcal organism combined with *Serratia marcescens* [5]. Despite this early promise, the mechanisms underlying bacterial therapy remain incompletely understood. The interactions between therapeutic bacteria and the host immune system are highly complex, and the metabolic byproducts and functional pathways of these microbes are not yet fully characterized. Current research has demonstrated that bacteria can influence tumor initiation, progression, and metastasis through multiple mechanisms, including the induction of pro-inflammatory microenvironments, metabolic reprogramming, and direct modulation of immune cell function [6,7]. One of the key advantages of bacteria lies in their ability to selectively colonize the hypoxic and immunosuppressive tumor microenvironment (TME), a feature that, when combined with their amenability to genetic engineering, positions them as a promising platform to overcome the limitations of conventional cancer therapies such as surgery, chemotherapy, and radiotherapy [8,9]. A notable example is the attenuated strain *Salmonella typhimurium* (VNP20009), which was evaluated in clinical trials for solid tumors [10]. However, the trial failed to demonstrate significant antitumor efficacy, largely due to excessive attenuation that impaired the bacteria's tumor-colonizing ability. This outcome underscores the critical need to balance attenuation with effective tumor targeting and immunogenicity. A more fundamental limitation arises from the inherent species differences between mice and humans in both the TME as well as the composition and function of the immune system. Compared with human tumors, murine tumors typically grow at a faster rate and display relatively uniform tissue architecture, which facilitates the formation of necrotic and hypoxic regions that serve as favorable niches for bacterial colonization. In contrast, human solid tumors are characterized by more complex stromal organization, vascular heterogeneity, and distinct patterns of immune cell infiltration, all of which not only restrict bacterial penetration and proliferation within the tumor but also accelerate bacterial clearance from the peripheral circulation [11]. In addition to biological challenges, technical barriers such as drug delivery, bacterial formulation, and administration strategies hinder the broader implementation of bacterial therapies in clinical settings. Therefore, the rational design of bacterial strains, along with the development of optimized delivery platforms, remains essential for enhancing both the efficacy and safety of bacteria-mediated cancer immunotherapy.

Recent advances in cancer immunotherapy have spotlighted the growing potential of nanomedicine as a transformative tool in enhancing therapeutic efficacy. Nanomaterials offer distinct advantages in the targeted delivery of immunomodulatory agents and genetic payloads to specific immune cell subsets, thereby amplifying systemic antitumor immune responses. Notably, certain nanomaterials possess inherent immunomodulatory properties, enabling them to directly modulate immune cell function, which can be divided into two categories in tumor immune regulation: acting on innate immunity and adaptive immunity [12]. Cationic nanocarriers, such as lipid nanoparticles (LNPs), synthetic polymers, and certain polysaccharides, enable efficient cytosolic delivery of mRNA, thereby promoting dendritic cell (DC) cross-presentation of tumor antigens and initiating antitumor immune responses. Reprogramming macrophages is another strategy to increase the responsiveness of immunologically “cold” tumors. For example, local injection of a hydrogel loaded with nanoparticles that continuously release a macrophage-specific-promoter-driven CAR plasmid targeting CD133 can induce adaptive immunity and reduce glioblastoma recurrence in mouse models [13]. Trispecific nanoengagers not only establish a physical bridge between tumors and NK cells but also amplify NK activity by engaging distinct activating pathways (anti-CD16 and anti-4-1BB), thereby eliciting immune-mediated tumor cytotoxicity [14]. In addition, nanomaterials can also be designed to re-energize T cell immunity. Nanogels can locally deliver agents that counteract immunosuppression (TGF-β inhibitors and IL-2) to restore T-cell function [15], while nano-scaffolds displaying anti-PD-1/anti-PD-L1 can physically couple T cells to tumor cells to promote antigen-specific cytotoxicity [16]. The structural versatility and engineering flexibility of these platforms further facilitate the rational design of multifunctional nanomedicines with customizable therapeutic features [17]. Beyond their delivery capabilities, nanomaterials exhibit a range of physicochemical properties, such as optical, magnetic, acoustic, and catalytic functionalities, that are particularly valuable in supporting bacteria-mediated cancer therapies [18]. The convergence of bacterial therapeutics and nanotechnology gives rise to synergistic nano-bacteria hybrids characterized by a dual mechanism of action: on one hand, bacteria exhibit natural tumor-targeting and colonization abilities that enhance the localized accumulation of nanodrugs within the TME; on the other hand, nanomaterials can modulate bacterial behavior or immune interactions to further amplify immunotherapeutic efficacy [[19], [20], [21]].

This review provides a comprehensive analysis of nano-bacteria hybrids in cancer immunotherapy, with a focus on three key areas: (1) the mechanisms by which bacteria mediate antitumor immune responses; (2) current strategies for engineering nano-bacteria hybrids that integrate bacterial vectors with nanotechnology; and (3) the innovative design and application of bacteria-inspired nano-bacteria hybrids for precision cancer immunotherapy. By elucidating the complex interplay between bacteria and the tumor immune microenvironment, and the role of nanotechnology in enhancing these interactions, this review aims to offer both theoretical insights and practical guidance for the development of next-generation intelligent immunotherapeutic platforms.

## 2 Bacteria in cancer treatment and a brief introduction of clinical trials

The therapeutic potential of bacterial immunotherapy stems from bacterial-derived PAMPs that engage Toll-like receptors (TLRs) on innate immune cells. This interaction induces robust activation of both CD4^+^ and CD8^+^ T cell populations through TLR-mediated signaling pathways [22]. While this innate immune activation provides broad pathogen alertness by triggering appropriate nonspecific defense mechanisms, its clinical application in oncology faces significant challenges due to the immunosuppressive TME. A critical barrier to effective cancer immunotherapy lies in the TME's capacity to subvert immune responses through increased infiltration of immunosuppressive cell populations. Remarkably, regulatory T cells (Tregs) and myeloid-derived suppressor cells (MDSCs) create an immunologically privileged niche that actively counteracts antitumor immunity, establishing a self-reinforcing cycle of immunosuppression [23]. Bacterial immunotherapy demonstrates dual functionality by simultaneously potentiating robust innate and adaptive antitumor immunity while modulating the tumor immune landscape through depletion of Tregs and associated immunosuppressive populations, thereby disrupting intratumorally immunosuppressive networks [24]. This immunomodulatory capacity stems from bacterial molecular signatures, including lipopolysaccharide (LPS) [25], peptidoglycan (PGN) [26], lipoteichoic acid (LTA) [27], and flagella [28], that mediate distinct immunomodulatory effects through PAMPs. Bacterial therapy activates both innate and adaptive immunity through the TLR signaling pathway while simultaneously remodeling the TME to break the cycle of immune suppression. This ability to coordinate multi-faceted immune activation forms the fundamental mechanism of bacteria-based anti-tumor strategies.

Bacteria exhibit unique advantages in tumor immunotherapy due to their natural tropism for the hypoxic tumor microenvironment, intrinsic immunogenicity, and genetic plasticity, making them ideal living drug delivery systems [29]. Obligately anaerobic genera (e.g. *Clostridium*, *Bifidobacterium*) and facultatively anaerobic species such as *Salmonella* can preferentially localize to and proliferate within hypoxic tumor cores. This behavior is driven by chemotactic responses to tumor-derived signals and by selective growth in low-oxygen niches, enabling these bacteria to bypass delivery barriers that limit many conventional therapies. Genetic engineering further enhances their capacity for precise immune modulation, and synthetic gene circuits enable both the amplification of immune interactions and the spatiotemporally controlled release of therapeutic agents. This living vector system achieves sustained in situ drug administration within tumors for the first time, addressing the pharmacokinetic limitations of traditional anticancer drugs. Currently, engineered bacteria-based immunotherapeutic strategies have entered clinical translation (Table 1), leveraging the natural tumor-targeting ability of bacteria and their immune-stimulating properties. *Salmonella Typhimurium*, *Listeria monocytogenes*, and *Clostridium novyi-NT* have been engineered to enhance antitumor immunity by delivering tumor-specific antigens or immune-modulating agents. The clinical trials of melanoma, cancer pancreatic cancer and lung cancer therapy have now progressed to Phase I and II trials.

**Table 1:** Clinical trials of bacteria-based cancer immunotherapy.

Species	Clinical trial identifiers	Strains	Engineered agents	Administration route	Trial phase	Number of patients
*Salmonella* Typhimurium	NCT00004988	VNP20009		Intravenous	Phase I	45
	NCT02718443	VXM01	Carrying VEGFR2-encoding DNA	Oral	Phase I	14
	NCT04589234	Saltikva	Containing human IL-2	Oral	Phase II	34
*Listeria monocytogenes*	NCT02002182	ADXS-HPV	Secreting tLLO-HPV-16 E7	Intravenous	Phase II	50
	NCT03847519	ADXS-503	Targeting 22 NSCLC-associated tumor antigens	Intravenous	Phase I- Phase II	Phase I: 9 Phase II: 17
	NCT01266460	ADXS11-001	Antigen: Human Papilloma Virus	Intravenous	Phase II	54
	NCT01675765	CRS-207	Antigen: mesothelin	Intravenous	Phase Ib	60
	NCT02592967	JNJ-64041757	Attenuated double deleted	Intravenous	Phase I	18
	NCT02592967	JNJ-64041809	Antigen: 4 antigens relevant to prostate cancer	Intravenous	Phase I	26
*Clostridium novyi-NT*	NCT01924689	C. novyi-NT	Lack of alpha toxin	Intratumoral	Phase I	24

Strain	Mechanism	Tumor Type	Safety	Efficacy and survival	Clinical advantages
VNP20009	Colonizes hypoxic/necrotic tumor regions; easy to engineer	24 patients with metastatic melanoma and 1 patient with metastatic renal cell carcinoma	Grade 3 AEs:7/24 Grade 4 AEs: none	Efficacy data were not clearly disclosed	Mechanism clear, platform suitable for immune factor delivery
VXM01	Induces VEGFR2-specific T cells to disrupt tumor vasculature	18 patients with locally advanced and stage IV pancreatic cancer	Mild fever, gastrointestinal discomfort; safe oral dosing Grade 3∼4AEs in Priming Phase:4/18 Grade 3∼4AEs in Boosting Phase:9/18	Efficacy data were not clearly disclosed	Oral delivery, systemic immune activation
Saltikva	Promotes innate/adaptive immunity via IL-2 release	34 patients with metastatic pancreatic cancer	No enrollment-related SAEs occurred.	Median PFS = 15 months Median OS = 20.3 months	Non-invasive administration, immunostimulatory potential
ADXS-HPV	Listeria monocytogenes expressing HPV-E7 antigen; APC targeting via LLO fusion	50 patients with platinum-refractory cervical carcinoma	Grade 3 AEs:19/50 Grade 4 AEs: 2/50	Median OS = 6.1 months (95 % CI: 4.3–12.1) Median PFS = 2.8 months (95 % CI: 2.6–3.0) ORR 6 % DCR 16 %	Validated HPV targeting; safe monotherapy option
ADXS-503	Listeria expressing tumor neoantigen cassette; enhances PD-1 response	26 patients with metastatic squamous or non-squamous non-small cell lung cancer	No detailed data were provided, AEs predominantly classified as grade 1–2, transient, and reversible.	ORR = 15.4 % DCR = 46.2 %	Effective in PD-1–resistant NSCLC; combination potential
ADXS11-001	Listeria-based HPV-E7 vaccine (similar to ADXS-HPV)	109 patients with advanced cervical cancer	SAE: 49/109	without Cisplatin: Median OS = 8.28 months (95 % CI: 5.85–10.5) Median PFS = 6.10 months ORR = 17.1 % DCR = 62.9 % With Cisplatin: Median OS = 8.78 months (95 % CI: 7.4–13.3) Median PFS = 6.08 months ORR = 14.7 % DCR = 58.8 %	Reproducible efficacy, clinical validation complete
CRS-207	Attenuated Listeria expressing mesothelin	35 patients with malignant pleural mesothelioma	Grade 3 AEs:4/35 Grade 4 AEs: none	Mesothelioma: ORR ≈ 12 %; Median PFS = 7.5 months (95 % CI, 7.0–9.9) Median OS = 14.7 months (95 % CI, 11.2–21.9) DCR = 89 %	Demonstrated antigen-specific immunity; moderate effect
JNJ-64041757	Double-deleted Listeria delivering tumor antigens	18 patients with with stage IIIB/IV NSCLC	SAE = 8/18	Without nivolumab: DCR = 44.44 % (8/18) With nivolumab DCR = 33.33 % (4/12)	Safe in combination with checkpoint inhibitors
JNJ-64041809	Listeria expressing PSA and other prostate antigens	26 patients with metastatic castration-resistant prostate cancer	SAE = 7/26	DCR = 52 %	Early-phase study; potential for prostate cancer immunotherapy
Clostridium novyi-NT	Anaerobic colonization of necrotic/hypoxic tumor regions; induces oncolysis and immune activation	24 patients with advanced injectable solid tumor	Grade 3–4 AEs: 5/24	ORR = 41 % DCR = 86 %	Strong hypoxia selectivity; rapid oncolysis with immune stimulation

Abbreviations: ORR = Objective response rate; PFS = Progression-free survival; OS = Overall survival; AE = Adverse event; DCR = Disease control rate. SAEs = serious AEs.

Clinical trial identifiers currently listed on ClinicalTrials.gov.

Bacterial therapy in cancer treatment faces two major challenges. First, achieving precise delivery of therapeutic payloads (such as chemotherapeutic drugs and immune modulators) to tumor sites while minimizing exposure to healthy tissues to avoid adverse side effects [30]. Second, overcoming the physical barriers of the TME, including hypoxia, acidity, and dense stroma, as well as biological barriers such as immune suppression and immune evasion, which hinder bacterial colonization and drug penetration [31]. These factors contribute to the clinical dilemma of balancing efficacy and safety in existing therapeutic strategies. Clinical trial outcomes have yielded important lessons for the future development of nano-bacteria hybrids. Early-phase clinical studies have focused on both signals of safety and efficacy. For example, the Phase I study of VNP20009 (an attenuated *Salmonella typhimurium*) showed that the agent could be administered safely at tolerable doses and that focal tumor colonization was observed in a very small number of patients. However, no objective tumor regression was seen, indicating that low colonization rates may limit antitumor efficacy. In addition, established microbial immunotherapies such as intravesical BCG have demonstrated clear clinical benefit owing to long clinical experience and the advantages of a local administration route (intravesical instillation). These are important points that engineered bacterial therapies should take into account when progressing toward broader clinical application.

The integration of nanotechnology with bacterial therapy represents a transformative approach in cancer treatment, addressing critical limitations of conventional immunotherapy through synergies. Nanomaterials serve as a beneficial tool for precision medicine due to their tunable surface properties, high drug-loading capacity, and ability to achieve targeted delivery [32,33]. Building on this foundation, nano-bacteria hybrids have emerged as multifunctional delivery platforms capable of transporting chemotherapeutics, phototherapeutic agents, and immunomodulators directly to tumor sites [34,35]. These hybrid systems leverage tumor microenvironment-specific triggers, such as acidic pH or elevated reactive oxygen species (ROS) levels, to enable spatially and temporally controlled drug release, significantly improving treatment precision [36,37]. By harmonizing the biological responsiveness of bacteria with the engineering versatility of nanomaterials, this approach establishes a novel paradigm for multimodal cancer treatment, demonstrating substantial potential to advance clinical outcomes and expand therapeutic applications in oncology.

## 3 Synergy between nanotechnology and bacterial therapy

**Fig. 1:**
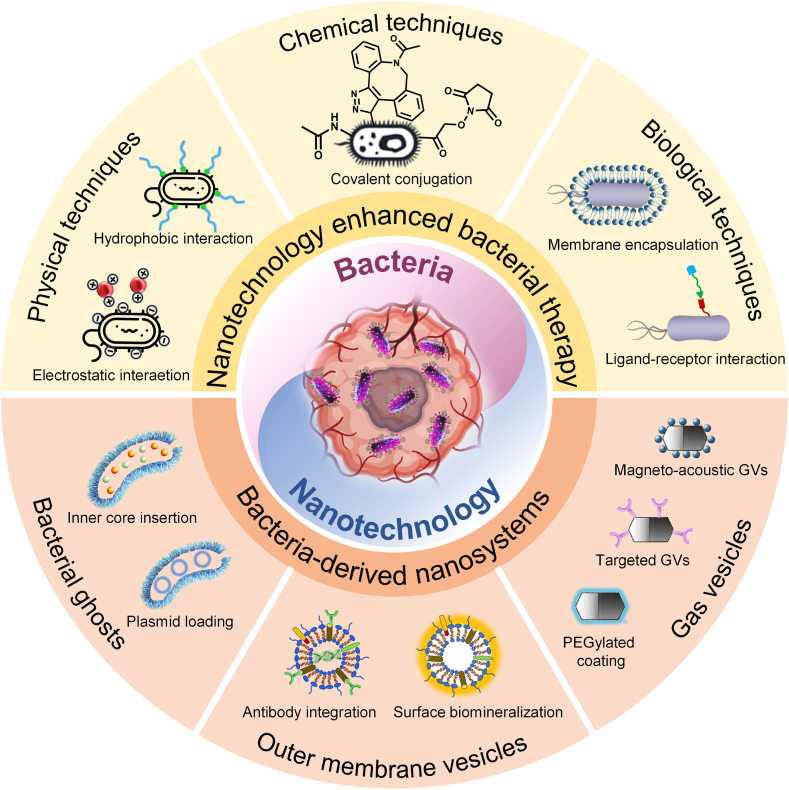
Schematic illustration of the integrated strategy for bacterial-nanotechnology synergy in constructing nano-bacteria hybrids, including the role of nanotechnology in enhancing bacterial therapy and the development of bacteria-derived nanoplatforms.

Leveraging their genetic programmability, rapid proliferation, and unique colonization advantages, bacteria have emerged as a hotspot in biomedical research. However, their inherent biotoxicity and insufficient colonization efficiency in diseased regions severely limit their clinical translation [38]. Notably, the abundant functional chemical groups on bacterial surfaces and their distinctive biological behavior in TME render them ideal platforms for functionalization. Accordingly, strategies such as reconstructing bacterial surface components and modifying their structure to enhance therapeutic efficacy while reducing toxicity, or introducing specific therapeutic units to impart new functions, have become key approaches in the development of bacterial therapies [39]. Currently, various physical, chemical, and biological methods are being explored to achieve efficient integration of nanomaterials with bacteria or to develop multifunctional nano-bacteria hybrids. These advanced hybrid systems hold great promises for improving treatment precision, minimizing side effects, and expanding the potential applications of bacterial therapies in cancer (Fig. 1). Table 2 summarizes the pros and cons of different interface binding strategies between nanomaterials and bacteria, including their *in vivo* stability, preservation of bacterial activity, and applicable scenarios. This comparative overview provides a clearer understanding of how different conjugation mechanisms influence biological performance and practical suitability in therapeutic applications.

**Table 2:** Pros and cons of interface binding strategy between nanomaterials and bacteria.

Combination strategy	Typical methods	Advantages	Disadvantages	Applicable scenarios
Physical strategy	1. Electrostatic interaetion 2. Hydrophobic interaction	1. A simple and convenient preparation method. 2. Causes minimal damage to bacterial viability. 3. Reversible loading facilitates controlled release.	1. Lower stability in complex physiological fluids. 2. Poor quantitative and spatial control; 3. Risk of nanoparticle aggregation altering surface properties.	1. Short-term *in vivo* delivery. 2. Applications prioritizing high bacterial viability.
Chemical strategy	1. Covalent conjugation	1. Strong and stable conjugation both in vitro and *in vivo*. 2. Precise control over conjugation sites and loading. 3. Suitable for long-term function.	1. Crosslinkers or harsh conditions may harm bacteria. 2. Process complexity and scale-up challenges. 3. Biosafety/regulatory concerns.	Applications requiring long-term stable delivery, imaging labeling, or adhesion maintenance.
Biological strategy	1. Membrane encapsulation 2. Ligand-receptor interaction 3. Biomineralization	1. Reaction under physiological conditions with minimal biological damage 2. Specific targeting of biomolecules 3. Excellent biocompatibility and biosafety	1. Poor controllability. 2. Issues with batch-to-batch heterogeneity and reproducibility. 3. Immunogenicity and biosafety risks.	Advanced applications requiring high-specificity targeting, long-term retention, or complex multifunctional integration.

### 3.1 Interface binding strategy between nanomaterials and intact bacteria

#### 3.1.1 Physical strategy

**Fig. 2:**
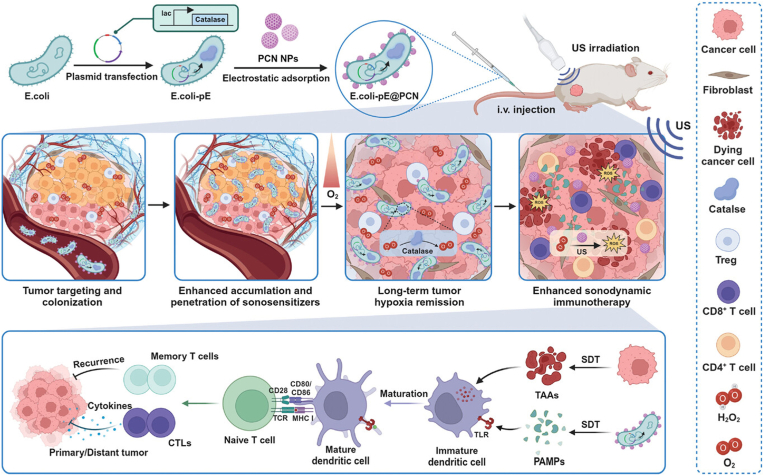
Scheme illustrating the programmable bacteria-based biohybrid, which was created by electrostatically attaching nano-sonosensitizers to engineered *E. coli*. After intravenous injection, the bacteria targeted and penetrated tumors, continuously producing catalase to relieve hypoxia and improving the accumulation of sonosensitizers. Sonodynamic therapy then triggered the release of tumor antigens and bacterial components, activating strong anti-tumor immune responses to inhibit tumor growth, metastasis, and recurrence [41]. Copyright 2024, John Wiley & Sons.

Cationic polymers or protonation strategies enable nanomaterials to reverse their surface potential from negative to positive, thereby facilitating electrostatic adsorption onto negatively charged bacterial surfaces [40]. Chen et al. [41] innovatively developed an engineered biohybrid system, termed *E. coli*-pE@PCN, by genetically modifying *Escherichia coli* to express catalase (*E. coli*-pE) and subsequently assembling it with positively charged nano-sonosensitizers (PCN NPs) via electrostatic interactions. This biohybrid forms the basis of a novel sonodynamic therapy (SDT) strategy. Engineered bacteria proliferate within tumors and continuously express catalase, functioning as a long-acting, self-sustaining in situ oxygen-generating system. This approach surpasses traditional enzyme delivery strategies by achieving sustained alleviation of tumor hypoxia. As living carriers, engineered bacteria actively migrate and colonize hypoxic tumor regions due to their anaerobic tropism, thereby enabling efficient enrichment of PCN NPs within the tumor. Leveraging their intrinsic motility, the bacteria can transport the associated nanoparticles beyond the perivascular region, facilitating active diffusion and deep penetration throughout the tumor parenchyma. These enhances the enrichment and intratumoral distribution of sonosensitizers. Importantly, this *E. coli*-pE@PCN-based SDT approach demonstrated potent therapeutic effects against both subcutaneous and orthotopic colorectal tumors. The treatment not only induced intense anti-tumor immune responses, mediated by the release of tumor-associated antigens (TAA) and PAMPs, but also generated durable immune memory, thereby preventing tumor recurrence. Furthermore, a notable abscopal effect was observed, effectively suppressing distant tumor metastases. Collectively, these results highlight the multidimensional anti-tumor potential of this engineered biohybrid SDT platform (Fig. 2).

Additionally, by leveraging the hydrophobic properties of the bacterial membrane bilayer, bioactive molecules with lipid-anchoring groups can be stably loaded through hydrophobic interactions. Zhu et al. [42] engineered a ROS-responsive cationic conjugated polyelectrolyte coating that stabilizes on *E. coli* surfaces via synergistic electrostatic and hydrophobic interactions. This dual-engineered bacterial system successfully integrates chemotherapeutic, photodynamic, and bacteriolytic modalities, demonstrating multimodal synergistic advantages in antitumor therapy.

#### 3.1.2 Chemical strategy

Physical adsorption strategies relying on electrostatic or hydrophilic/hydrophobic interactions have been widely employed for the fabrication of nano-bacteria hybrids due to their simplicity. However, the presence of abundant proteins, salts, and other competing biomolecules *in vivo* often compromises the interfacial stability. In comparison, covalent coupling provides a more robust linkage between bacterial surfaces and nanomaterials under complex physiological conditions. The feasibility of covalent modification is attributed to the diverse chemical functionalities naturally presented on bacterial envelopes, which are composed of polysaccharides, proteins, and lipids. Reactive moieties such as amino (–NH_2_), carboxyl (–COOH), and thiol (–SH) groups provide versatile anchoring sites. For example, surface amines readily react with N-hydroxy succinimide (NHS) esters to form stable amide bonds, with isocyanates to yield urea linkages, or with aldehydes to generate Schiff bases [43]. Similarly, carbodiimide reagents such as EDC activate carboxyl groups for conjugation with amino-bearing ligands, including antibodies or aptamers, thereby endowing bacteria with enhanced targeting capacity. Thiol–Michael addition has emerged as an efficient strategy for covalent functionalization of bacterial surfaces by exploiting the free thiol groups (−SH) present in cysteine residues of surface proteins. These thiols, owing to their strong nucleophilicity, readily undergo nucleophilic addition with α,β-unsaturated carbonyl compounds to generate stable thioether bonds, thereby providing a robust means of anchoring functional moieties onto bacterial membranes. Notably, maleimide-modified upconversion nanoparticles can be conjugated with thiolated bacteria under mild physiological conditions [44]. Surface engineering strategies based on covalent conjugation allow precise modulation of these biological behaviors. On one hand, the application of “stealth” coatings effectively masks highly immunogenic components such as LPS, thereby reducing premature clearance by the immune system during blood circulation and improving the delivery efficiency of viable bacteria to tumor sites. On the other hand, covalent connections of tumor specific targeting parts can achieve active and precise identification and colonization of lesion areas, which is superior to innate tendencies [45]. Conventional photosensitizer-based therapies often face limitations such as unitary and easily attenuated effects, poor tumor penetration and retention, and the requirement for multiple irradiations in combination therapies. To address these challenges, the pioneering work by Liu et al. [46]. integrates synthetic chemistry with synthetic biology to develop a bacteria-based, monochromatically excitable multifunctional platform for phototheranostics. The core strategy involves leveraging the biomimetic deposition properties of polydopamine to achieve efficient integration and functional synergy of photosensitizers on the surface of engineered bacteria.

**Fig. 3:**
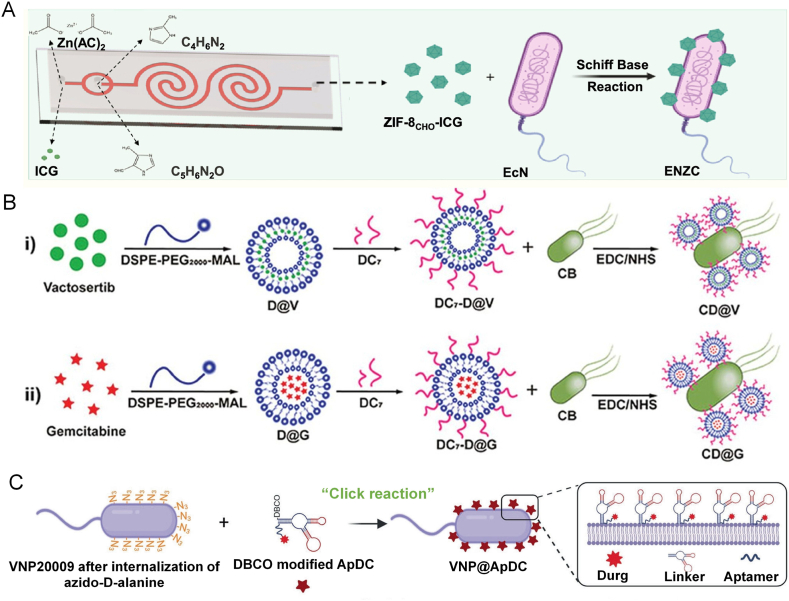
(A) Synthesis of EcN-ZIF-8CHO-ICG through Schiff base reaction: an ENZC composite was constructed by coating engineered EcN (expressing anti-PD-L1/CD9 nanobodies) with ICG-loaded ZIF-8 MOF for spatiotemporally controlled bacterial lysis and immunotherapy [47]. Copyright 2024, American Chemical Society. (B) Schematic illustration of preparation process of the MMP-2 responsive probiotic-nanosystem: drug-loaded liposomes were first conjugated with an MMP-2-cleavable peptide (DC7) via a maleimide-sulfhydryl reaction, and then covalently linked to Clostridium Butyricum (CB) via an EDC/NHS reaction to form the probiotic-nanosystem [48]. Copyright 2024, John Wiley & Sons. (C) Schematic flow depicting the functionalization of bacteria: anchoring ApDC to VNP20009 using a simple one-step click chemistry process [50]. Copyright 2024, Springer Nature Limited.

For instance, Song et al. [47] utilized microfluidic technology to fabricate indocyanine green (ICG) loaded metal organic framework (ZIF-8CHO) nanoparticles. They were conjugated onto the surface of *Escherichia coli* Nissle 1917 (EcN-Nb) co-expressing anti-PD-L1/CD9 nanobodies via Schiff base reactions, forming an engineered ENZC hybrid system. This system effectively reverses tumor-induced immunosuppression by targeting the PD-L1/CD9 dual pathway, thereby inhibiting tumor exosome-mediated migration. It demonstrated significant efficacy in promoting tumor necrosis and suppressing lung metastasis (Fig. 3A). Similarly, Zhang et al. [48] employed an EDC/NHS coupling reaction to conjugate modified liposomes with *Clostridium butyricum* (CB), rationally developing an engineered probiotic–nanoparticle system endowed with MMP-2 responsiveness and tumor-targeting capabilities. This system follows a "stepwise" strategy to enhance gemcitabine-induced chemoimmunotherapy against pancreatic tumors: the first step (CD@V) remodels the TME by suppressing pancreatic stellate cells and extracellular matrix production, while the second step (CD@G) enhances deep drug penetration and potency by killing tumor cells and preventing gemcitabine degradation (Fig. 3B). Considering the overexpression of CD44/CD168 receptors in colorectal tumors, Huang et al. [49] utilized the condensation reaction between carboxyl groups from gram-negative bacterial N-acetylmuramic acid and hydroxyl groups from hyaluronic acid (HA) to construct an HA-coated bacterial delivery system. This approach enables bacteria to withstand harsh gastrointestinal environments while achieving targeted accumulation at colorectal tumor sites, offering a promising avenue for cancer immunotherapy through oncolytic microbial-programmed cell death. Furthermore, Tan et al. [50] employed a click chemistry strategy to covalently attach a DBCO-modified aptamer–drug conjugate (ApDC), Sgc8c-MMAE, to the surface of azide-functionalized Salmonella strain *VNP20009*. This design combines the tumor targeting and penetrability of the bacteria with the specific recognition and cytotoxicity of the aptamer-drug conjugate. Under anaerobic conditions, the engineered bacteria actively home to pancreatic tumors and bind to tumor cells through aptamer-mediated interactions, offering a synergistic and targeted approach for effective pancreatic cancer therapy (Fig. 3C).

#### 3.1.3 Biological strategy

Physical and chemical methods can modify bacteria by exploiting the negative charge or functional groups on their surfaces, thereby endowing them with various exogenous properties. In contrast, bioengineering techniques can introduce more persistent functionalities. Biological strategies, employing mechanisms such as membrane fusion or molecular recognition, enable the fabrication of nano-bacteria hybrids that minimize bacterial damage while preserving their intrinsic therapeutic capabilities.

**Fig. 4:**
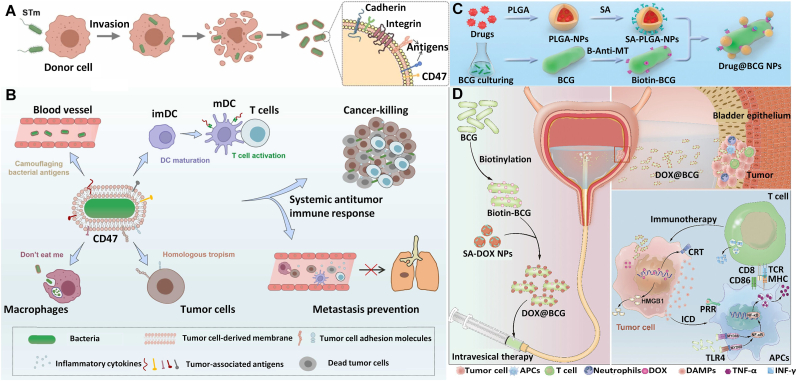
(A) Multimodal oncolytic bacteria are prepared by encapsulating tumor cell nanochells: upon ultraviolet (UV) irradiation, the intracellularly pre-colonized bacteria are automatically encapsulated by an extra membrane derived from apoptotic bodies (ABs) generated by the infected tumor cells. (B) Under external stimulation, bacteria invade tumor cells and induce apoptosis, forming an additional envelope and enhancing the ability of microbial therapy of tumors [51]. Copyright 2022, Elsevier B.V. (C) A live BCG-based drug delivery system, the DOX@BCG complex, was developed by conjugating DOX nanoparticles to the BCG surface through the robust biotin-streptavidin binding system. (D) After bladder administration, DOX induces ICD in tumor cells, while the combination of BCG PAMPs and DAMPs produced by ICD activates immune cells, achieving a synergistic anti-tumor effect [53]. Copyright 2024, John Wiley & Sons.

Surface-associated immunogenicity and pathogenicity have significantly hindered the clinical translation of bacterial therapies, particularly for systemic administration. Although various attenuated bacterial strains have been employed as alternatives, these species still exhibit dose-dependent side effects. Given the superior biocompatibility of natural cell membranes, cell membrane coating techniques have been applied to bacterial surfaces to protect them from immune clearance and enhance their therapeutic efficacy. For example, Liu et al. [51] developed a cancer cell-mimicking bacteria (CCMB) system using a biomimetic membrane coating technique. In this system, attenuated bacteria are encapsulated with tumor cell–derived nanomembranes to form a biotherapeutic agent with dual treatment modalities. Under external stimulation, the pre-encapsulated bacteria are spontaneously cloaked by an additional membrane layer derived from apoptotic bodies of infiltrating tumor cells. As a result of the tumor cell–derived membrane, the CCMB exhibits reduced inflammatory responses, slower systemic clearance, homologous tumor targeting, and a dual-mode therapeutic effect that combines antigen-specific immunotherapy with microbial therapy (Fig. 4A and B).

Streptavidin, a term encompassing both streptavidin and its homologs, is extensively utilized in molecular science, thanks to its highly specific and stable interaction with biotin [52]. Zhao et al. [53] engineered a synergistic chemo-immunotherapy platform by leveraging the natural tropism of Mycobacterium bovis Bacillus Calmette–Guérin (BCG) for bladder epithelium. They developed DOX@BCG, a biohybrid complex constructed through a biotin–streptavidin conjugation system to stably anchor doxorubicin (DOX)-loaded nanoparticles onto the BCG surface (Fig. 4C). This design capitalizes on BCG's intrinsic ability to adhere to and infiltrate bladder tumor cells, enabling spatially controlled delivery of chemotherapeutic payloads while amplifying intratumoral drug accumulation. Upon intravesical administration, DOX@BCG adheres to the bladder epithelium and is internalized by tumor cells, where pH-sensitive nanoparticle degradation triggers DOX release. The liberated DOX induces nuclear DNA damage-mediated chemotherapeutic cell death and immunogenic cell death (ICD), characterized by the release of damage-associated molecular patterns (DAMPs) such as ATP and HMGB1. Concurrently, BCG-derived PAMPs, including mycobacterial cell wall components, synergize with DAMPs to activate antigen-presenting cells (APCs) within the tumor microenvironment. This dual signaling cascade promotes dendritic cell maturation and tumor antigen cross-presentation to CD8^+^ cytotoxic T lymphocytes (CTLs), while also recruiting pro-inflammatory immune subsets—including M1-polarized macrophages, neutrophils, and effector T cells, into tumor beds. Preclinical evaluation in orthotopic bladder cancer models demonstrated that DOX@BCG not only suppressed primary tumor progression but also extended survival in treated rodents (Fig. 4D). These findings position DOX@BCG as a transformative approach for bladder cancer treatment, merging precision chemotherapy with in situ vaccination effects to overcome conventional limitations of standalone therapies.

### 3.2 Nanoplatforms constructed using bacterial components

Nano-bacteria hybrids, including outer membrane vesicles (OMVs), bacterial ghosts (BGs), and gas vesicles (GVs), have emerged as promising candidates in nanomedicine due to their inherent bioactivity, biocompatibility, and engineering versatility. These acellular particles, ranging from sub-100 nm to micrometer scales, exhibit structural heterogeneity distinct from uniformly assembled synthetic nanomaterials. Despite lacking genomic DNA for replication, they retain functional bacterial components, enabling precise drug delivery, immunomodulation, and diagnostic applications. Their unique capacity to mimic natural biological interactions while avoiding replicative risks positions them as transformative tools in therapeutic and diagnostic innovation.

#### 3.2.1 Outer membrane vesicles (OMVs)

**Fig. 5:**
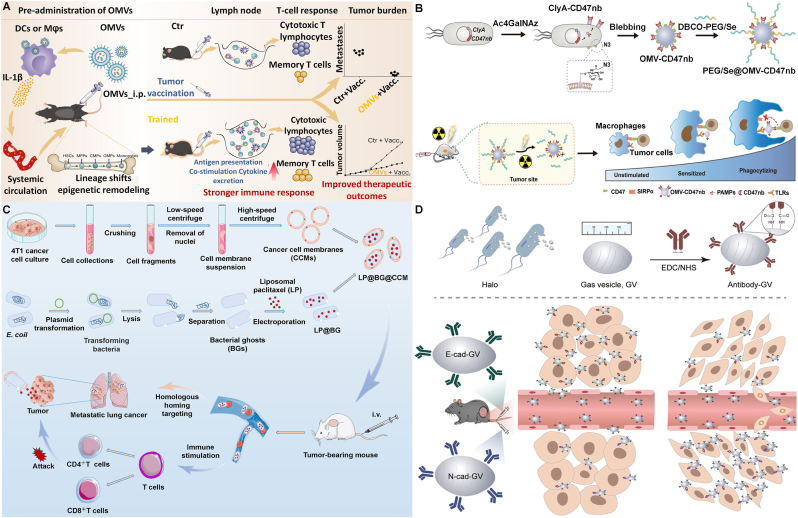
(A) OMV-induced trained immunity remodels bone marrow hematopoietic stem and progenitor cells via IL-1β signaling, enhancing the function of peripheral immune cells and thereby significantly boosting antitumor responses following tumor vaccination [56]. Copyright 2023, Springer Nature Limited. (B) The schematic illustrates the construction of a controllable two-way adaptor, PEG/Se@OMV-CD47nb, by displaying ClyA-CD47 nanobody on OMV surfaces and introducing azide groups for click reaction with DBCO-modified PEG/Se, ultimately enabling multipath activation of macrophage phagocytosis [57]. Copyright 2022, John Wiley & Sons. (C) Paclitaxel was first encapsulated into liposomes (LP), then the LP was encapsulated into BGs to form LP@BG, and ultimately encapsulated within CCMs to yield the final LP@BG@CCM [63]. Copyright 2024, Elsevier B.V. (D) Design of biosynthetic GVs for tumor EMT monitoring: E-cad-GVs and N-cad-GVs were fabricated by conjugating anti-E-cadherin or anti-N-cadherin antibodies to biosynthetic GVs via EDC/NHS crosslinking [70]. Copyright 2024, John Wiley & Sons.

OMVs are spherical, bilayered nanostructures (20–250 nm) released from the cell membranes of gram-negative bacteria, containing various immunostimulatory components such as virulence factors, enzymes, bacteria-specific antigens, and PAMPs [54]. Multiple studies have confirmed that bacterial OMVs can accurately target tumor sites and accumulate, thereby inducing the expression of anti-tumor cytokines such as CXCL10 and interferon-γ [55]. Nie et al. [56] demonstrated that administration of PAMP-enriched bacterial OMVs prior to vaccination can enhance tumor vaccine efficacy by inducing immune training. Notably, the nanoparticulate nature of OMVs, compared to free LPS, was key to their superior immunomodulatory effect, ultimately boosting the therapeutic efficacy of tumor vaccines (Fig. 5A).

In addition to their natural biological functions, OMVs can be engineered via antibody integration and surface mineralization to achieve controlled drug release and improved biosafety. Zhao et al. [57] first engineered OMVs by fusing a neutralizing CD47 nanobody (CD47nb) to the surface protein ClyA, generating OMV-CD47nb. These vehicles were then further coated with a diselenide bond-containing layer. Acting as a dual-targeting bridging protein, OMV-CD47nb simultaneously binds to CD47 on tumor cells and to TLR on tumor-associated macrophages (TAMs). This dual interaction not only promotes M1 polarization of TAMs but also blocks the “don't eat me” signal on tumor cells. Furthermore, the TAM-mediated phagocytosis of tumor cells triggered by OMV-CD47nb induces the release of tumor antigens, which are subsequently processed by APCs and presented in the draining lymph nodes, ultimately eliciting a T cell-mediated antitumor immune response (Fig. 5B). Considering that PAMPs in OMVs can cause systemic toxicity and hinder their clinical translation, Hu et al. [58] employed a genetic engineering strategy to produce OMVs loaded with melanin. These OMVs were then surface mineralized with calcium phosphate (CaP) to form OMV^Mel^@CaP, thereby reducing toxicity. The CaP encapsulation significantly diminishes systemic inflammatory responses during circulation and further alleviates liver damage induced by OMV^Mel^. Upon accumulation in the tumor region, the CaP shell disintegrates in the acidic TME, exposing the OMV^Mel^ to trigger an antitumor immune response through the induction of dendritic cell maturation and consequent T cell activation.

#### 3.2.2 Bacterial ghosts (BGs)

BGs represent empty cellular envelopes formed when Gram-negative bacteria undergo controlled lysis via PhiX174 gene E expression [59]. BGs retain the bacterial cell morphology and surface structures, containing various bacterial components such as lipopolysaccharides and lipoproteins, along with peptidoglycan and outer membrane proteins. This enables them to offer excellent targeting abilities and adjuvant properties, making them a potentially safe alternative to toxic bacteria [60,61]. The inherent particle architecture and surface properties of BGs enable their spontaneous targeting to primary APCs when used as delivery carriers. Antigens, recombinant proteins, or DNA can be associated with the inner membrane, outer membrane, or periplasmic space of BGs, supporting flexible antigen presentation strategies [62].

With their large internal space and intact outer wall, BGs can accommodate various payloads. Jin et al. [63] developed a layer-by-layer encapsulation strategy, sequentially packaging paclitaxel in liposomes, BGs, and cancer cell membranes to prepare LP@BG@CCM. By ingeniously coating paclitaxel-loaded bacterial BGs with homologous cancer cell membranes, the biomimetic system addresses both efficacy and safety challenges in metastatic lung cancer therapy. This "two-birds-one-stone" strategy leveraged the BG's large size for lung retention and the membrane's homing ability for tumor targeting, while the outer membrane also served to temper the BG's inherent strong immunogenicity, preventing excessive inflammation (Fig. 5C). To address the challenge of limited motility in BGs-based delivery systems, Li et al. [64] employed electroporation to successfully co-loaded hydrophilic 5-fluorouracil (FU) and hydrophobic zoledronic acid (ZOL) into the probiotic *Escherichia coli* Nissle 1917 (EcN) and subsequently modified the surface of EcN with gold nanorods (Au NRs). Li et al. [65] designed a new-type Neisseria gonorrhoeae DNA vaccine delivered via BGs. Compared to using the Neisseria gonorrhoeae DNA vaccine alone, the oral BGs vaccine elicited stronger CD4^+^ and CD8^+^ T cell responses and promoted higher IgG levels. BGs may act as vaccine adjuvants by promoting the maturation of BMDCs, which subsequently amplifies the vaccine antigen-specific immune response.

#### 3.2.3 Gas vesicles (GVs)

Novel biologically derived gas vesicles (GVs) are nanometer-scale vesicles found in certain planktonic microorganisms, such as halophilic archaea and cyanobacteria [66]. They consist of a gas-filled core encapsulated by a protein shell, typically measuring 45–250 nm in width and 100–600 nm in length, with terminal structures that are either conical or spindle-shaped. Shapiro MG and colleagues were the first to apply GVs from halophilic archaea and cyanobacteria for ultrasound imaging, demonstrating that these vesicles offer excellent contrast performance. [67]. The heterologous expression of engineered genetic clusters encoding GVs enables *Escherichia coli* and *Salmonella typhimurium* to achieve non-invasive imaging at a volume density below 0.01 % with a resolution of less than 100 μm. This allows microbial cells to be visualized deep within mammalian hosts, facilitating research on the mammalian microbiome and the development of diagnostic and therapeutic cell-based formulations [68].

The surface modification with polyethylene glycol (PEG) significantly reduces the uptake of nanoparticles by the reticuloendothelial system (RES) and prolongs their circulation time *in vivo*. Additionally, PEG coating can shield the surface antigens of nanoparticles, minimizing immune responses. Yan et al. [69] modified the biosynthesized GVs of Halobacterium NRC-1 with PEG to prepare PEG-GVs. The PEG-GVs exhibited lower clearance by RES macrophages and a longer circulation time, resulting in improved contrast imaging *in vivo*.

Compared to synthetic vesicles, GVs have abundant active functional groups on their surface, making them easier to functionalize and an excellent candidate for targeted delivery of therapeutic molecules to cells or tissues. Yan et al. [70] developed dual-targeted nanobubble system constructed by covalently linking both E-cadherin-specific and N-cadherin-specific antibodies to the surface of genetically modified GVs, enabling noninvasive tracking of epithelial–mesenchymal transition (EMT) biomarkers. These nanobubbles were designed to monitor the dynamic expression of E-cadherin (epithelial marker) and N-cadherin (mesenchymal marker) during tumor progression, a key indicator of metastatic potential. Using ultrasound molecular imaging, the system demonstrated high specificity and sensitivity in detecting real-time shifts in cadherin profiles within tumor cells. This approach offers a promising tool for visualizing EMT dynamics, facilitating early evaluation of tumor aggressiveness and treatment response (Fig. 5D). In addition, Kim et al. [71] engineered magnetic gas vesicles (MGVs) by integrating superparamagnetic nanoparticles (MNPs) with GVs, creating a novel hybrid nanostructure. This MNP-GV conjugation demonstrates superior signal intensity and sensitivity compared to traditional MMUS contrast agents. The remarkable stability of MGVs renders them particularly suitable for prolonged monitoring in fibrotic disease models and high-content screening using 3D organoids, providing consistent imaging performance.

## 4 Integration of nanotechnology and bacterial therapy in precise immunotherapy

### 4.1 Nanotechnology based bacterial therapy and immune checkpoint therapy

**Fig. 6:**
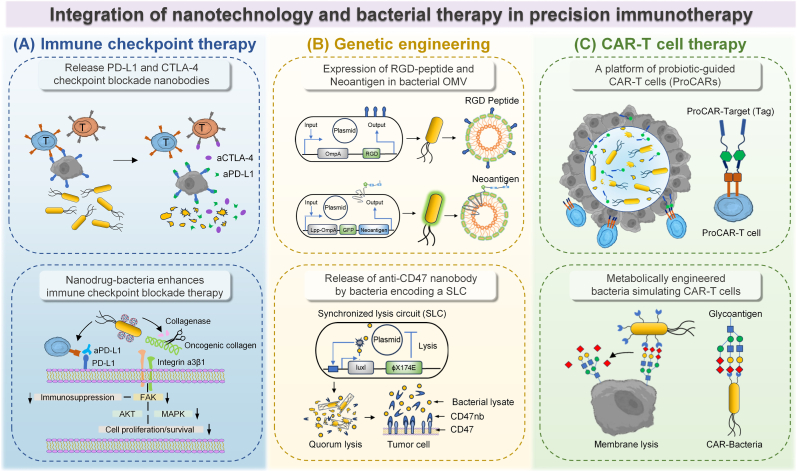
The application of nano-bacteria hybrids in precision immunotherapy. (A) Probiotic-mediated controlled delivery of anti-PD-L1 and CTLA-4 nanobodies to tumors via stabilized lytic release [74]. Protein cages composed of collagenase and aPD-L1s were attached to ECN surface, forming Col/aPD-L1@ECN [75]. (B) RGD-expressing bacterial double layer membrane-derived nanovesicles (DMVs) with endogenous targeting ligands form this drug delivery system [88]. Engineered bacteria synthesized fusion neoantigens, yielding bacteria derived vesicles (BDVs) that displayed the antigens [91]. Modified non-pathogenic *E. coli* lyses in TME to release CD47-antagonist nanobody [92]. (C) Engineered bacteria detect tumor-associated sialic acid, locally produce cytolysin to lyse cancer cells, and thereby simulate CAR-T cell functionality [103]. Probiotic-guided CAR-T cell (ProCAR) platform utilizes tumor-colonizing probiotics to release synthetic markers that specifically label tumor tissue for in situ CAR-mediated lysis [104].

Recent advances propose the synergistic integration of nano-bacteria hybrids with ICB (Fig. 6A). Genetically engineered bacterial vectors facilitate tumor-specific delivery and sustained release of checkpoint inhibitors, thereby minimizing the off-target effects associated with conventional drug delivery systems. This multimodal approach integrates spatiotemporally controlled drug release with immune microenvironment modulation, offering a promising avenue for advancing precision cancer immunotherapy.

Bi et al. [72] utilized two types of bivalent and trivalent nanobodies targeting the SIRPα/CD47 and PD-L1/PD-1 pathways. The nanobodies were site-specifically immobilized on OMVs via two orthogonal protein conjugation systems: SpyCatcher/SpyTag and SnoopCatcher/SnoopTag, generating an OMV-based dual-functional nanoscale immune cell engager (OMV-NICE). This construct enhanced tumor cell binding while recruiting and activating macrophages and T cells both in vitro and *in vivo*. In addition to rapid exogenous blockade through direct PD-L1 binding, Zhou et al. [73] introduced a long-term endogenous therapeutic approach based on LyP1 peptide-modified OMVs (LOMVs) carrying PD-1 plasmids, which induced self-sustained PD-L1 blockade in tumor cells. The nanocarrier selectively accumulated in tumor tissues through OMV-mediated targeting and was internalized by tumor cells via LyP1-driven endocytosis. Subsequently, the PD-1 plasmid was successfully delivered into the nucleus, triggering sustained PD-1 expression in tumor cells. The outer membrane proteins of LOMVs facilitated the recruitment of cytotoxic T lymphocytes (CTLs) and natural killer (NK) cells into the TME, where these immune cells were stimulated to secrete interferon-gamma (IFN-γ), ultimately potentiating the antitumor effect of the PD-1/PD-L1 self-blockade therapy.

**Fig. 7:**
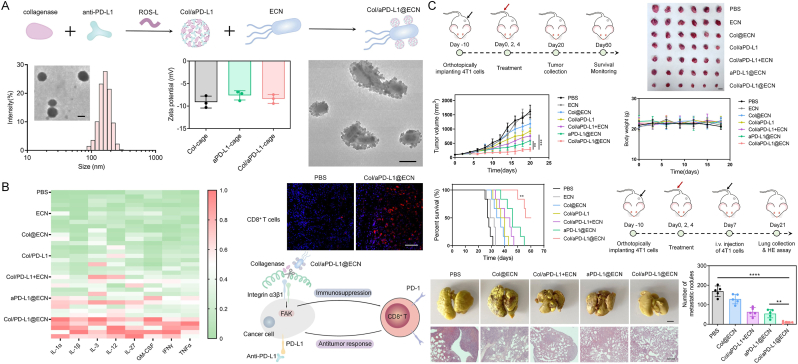
(A) A protein cage formulation composed of collagenase and anti-PD-L1 antibodies (aPD-L1s) was constructed using a ROS-responsive linker. This formulation was then conjugated to the surface of the probiotic ECN to form Col/aPD-L1@ECN. (B) Immune profiling of Col/aPD-L1@ECN therapy: Luminex-based cytokine quantification (n = 5), immunofluorescence analysis of CD8^+^ T cell infiltration (scale bar = 100 μm), and schematic of the proposed immune activation mechanism. (C) Antitumor efficacy evaluation of Col/aPD-L1@ECN in a metastatic breast cancer model [75]. Copyright 2024, Elsevier B.V.

Beyond the use of bacteria, derived OMVs for immune checkpoint therapy, engineered bacteria have also been employed for the localized tumor delivery of checkpoint-blocking nanobodies. Danino et al. [74] developed a probiotic-based delivery system that enabled controlled production and intertumoral release of PD-L1- and CTLA-4-targeting nanobodies through a stable bacterial lysis mechanism. In mice treated with checkpoint inhibitors delivered by probiotics, activated T cells increased significantly, leading to an abscess-like effect and a corresponding expansion of systemic T cell memory populations. This engineered probiotic system integrated synthetic biology with immunology, improving the delivery of checkpoint blockade therapies. As an alternative strategy for integrating immune checkpoint blockade with bacterial systems, Hu et al. [75] developed a bacteria-based protein drug delivery system targeting pancreatic ductal adenocarcinoma (PDAC), leveraging the hypoxia-targeting capability and motility of the probiotic *Escherichia coli* strain Nissle 1917 (ECN). By loading ECN with a collagenase and anti-PD-L1 conjugate (Col/aPD-L1@ECN), the treatment significantly enhanced cytokine expression and promoted CD8^+^ T cell infiltration within PDAC tumors, indicating a robust antitumor immune response. This therapeutic strategy demonstrated significant efficacy in a collagen-rich 4T1 metastatic breast cancer model, with Col/aPD-L1@ECN treatment achieving both substantial suppression of orthotopic tumor growth and extension of median survival to 58 days, with minimal impact on body weight—suggesting good biosafety. In a lung metastasis model, the treatment also significantly reduced both the number and size of metastatic nodules. Mechanistic investigations revealed that reactive oxygen species (ROS) present within the PDAC microenvironment orchestrated the enzymatic liberation of collagenase, which subsequently mediated: proteolytic cleavage of tumor-promoting collagenous matrices, abrogation of integrin α3β1-FAK-mediated oncogenic signaling transduction, and mitigation of immunosuppressive tumor niche characteristics. This tripartite mechanism collectively ameliorated tumor-mediated immune suppression, thereby synergistically augmenting the therapeutic efficacy of PD-L1 immune checkpoint inhibition (Fig. 7).

### 4.2 Nanotechnology based bacterial therapy and genetic engineering

Synthetic biology has achieved significant advances in engineering genetic circuits and precisely controlling biological functions, particularly offering innovative solutions for cancer therapy [76]. By identifying and manipulating gene clusters, thereby achieving precise control over cellular behavior. Bacteria, as an essential chassis in synthetic biology, have been genetically engineered into promising tools for tumor therapy [77,78] (Fig. 6B). The core of genetic engineering lies in the rational design of genetic circuits, enabling bacteria to sense and respond to the complexities of the tumor microenvironment as well as some exogenous stimuli [[79], [80], [81]]. Boolean logic-gated circuits can be designed to facilitate multi-modal combination therapies [82], while synchronized lysis mechanisms allow for controlled drug release, preventing premature diffusion and deactivation before reaching the tumor site, thereby enhancing bacterial targeting specificity [83]. Additionally, engineered bacteria have been endowed with the capability to express and selectively deliver therapeutic molecules. Our team has developed an in situ probiotic vaccine (FOLactis) based on the food-grade probiotic *Lactococcus lactis*, engineered to express a fusion protein consisting of Fms-like tyrosine kinase 3 ligand (Flt3L) and the co-stimulatory molecule OX40 ligand (OX40L). FOLactis has demonstrated enhanced antitumor immunity and effectively converted "cold" tumors into "hot" tumors [84].

**Fig. 8:**
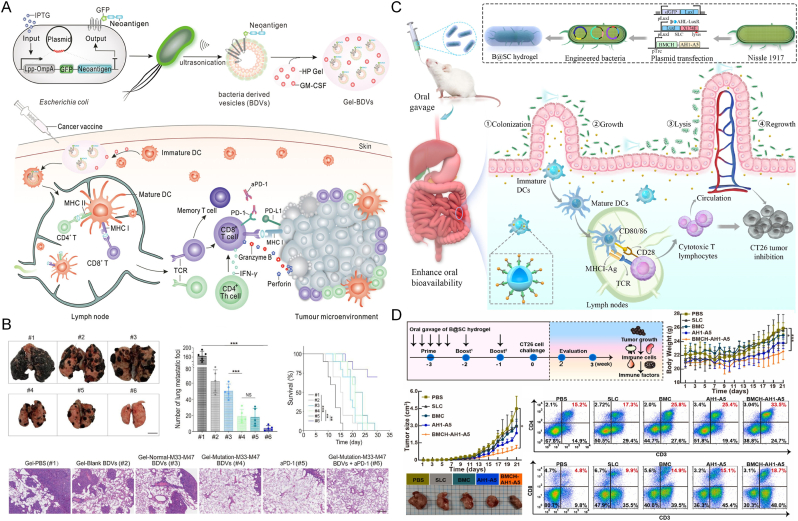
(A) Melanoma-targeting BDVs (Gel-GFP-Mutation-M33-M47) combined with PD-1 blockade for melanoma immunotherapy: fabrication, T cell activation, and therapeutic effects. (B) Treatment outcomes in melanoma lung metastasis: metastasis inhibition, survival, and lung histology [91]. Copyright 2022, John Wiley & Sons. (C) Engineered oral bacterial hydrogel produces nano-vaccines in situ via SLC circuit, colonizes gut, and activates immunity against tumors. (D) BMCH-AH1-A5 nano-vaccine efficacy against CT26 tumors: experimental design, tumor/weight monitoring, and CD4^+^/CD8^+^ T cell activation in lymph nodes [93]. Copyright 2023, Elsevier B.V.

Strategies to enhance bacterial tumor specificity include mutagenesis and directed evolution, often selecting nutrient-auxotrophic bacterial strains that preferentially colonize tumor tissues [85], or engineering bacteria to express tumor-targeting components, such as adhesion peptides or tumor-associated antigens, on their surface [86]. For example, Min et al. [87] have fused adhesion peptides with outer membrane protein A (OmpA) to engineer an attenuated *Salmonella* strain that displays the tumor-homing arginine-glycine-aspartic acid (RGD) peptide. RGD subsequently binds to αvβ3 integrins, which are widely overexpressed on tumor cells, significantly enhancing bacterial adhesion to tumor cells compared to healthy cells. A similar approach has been applied to bacterial OMVs, where RGD peptides are displayed on the vesicle surface to enable simultaneous targeting of multiple cell populations, including neutrophils, monocytes, and both tumor vasculature and cells. Ultimately, this improved the accumulation of tumor specific DOX [88]. Beyond expressing tumor-targeting peptides, bacteria can also be engineered to display tumor-associated antigens [89,90]. Zhang et al. [91] genetically created a novel platform where engineered bacteria biosynthesized tumor neoantigens, which were then naturally presented on bacterial-derived vesicles (BDVs-Neo) as customizable therapeutic vaccines (Fig. 8A). The study evaluated the efficacy of Gel-Mutation-M33-M47 BDVs combined with anti-PD-1 antibody (aPD-1) in a lung metastasis model. Mice treated with the combination therapy showed significantly fewer metastatic nodules and an improved survival rate of approximately 70 %. Analysis of lung tissues fixed with 4 % paraformaldehyde confirmed fewer metastatic lesions in groups receiving either BDVs or aPD-1 alone, with the combination group exhibiting the most pronounced suppression of metastasis. H&E staining validated the marked inhibition of metastatic progression. Mechanistically, Gel-BDVs-Neo binds to the PD-1 antibody, enhancing the multiplication and phenotypic activation of tumor-infiltrating T cells and promoting the expansion of memory T cell clones. These results underscore the strong synergistic potential of Gel-Mutation-M33-M47 BDVs and aPD-1 in controlling tumor metastasis (Fig. 8B).

Genetically engineered bacteria can release recombinant immunotherapeutic agents in a controlled manner in tumor microenvironments, achieving higher local effective therapeutic concentrations while avoiding toxicity associated with systemic delivery of similar drugs. Danino et al. [92] designed a non-pathogenic strain of *E. coli* containing a synchronized lysis circuit (SLC), which undergoes TME-responsive lysis to precisely deliver the CD47-targeting nanobody (CD47nb). Local administration of CD47nb-expressing engineered bacteria induces systemic tumor antigen-specific immunity, mediating abscopal suppression of distal tumor growth. It provided conceptual validation for the "abscess effect" induced by engineered bacterial immunotherapy. In addition, Wang et al. [93] developed a programmable oral bacterial hydrogel system for precise vaccine delivery. To enhance gastric survivability, engineered bacteria were microencapsulated in a chitosan–alginate matrix while being genetically modified to express tumor antigen-presenting nano-vaccines. Controlled release was achieved through a synthetic biological SLC circuit (Fig. 8C). In vivo studies showed that the BMCH-AH1-A5 treatment group exhibited the smallest tumor volume, indicating effective immune-mediated tumor suppression. Additionally, 21 days after tumor inoculation, mice treated with BMCH-AH1-A5 showed significantly elevated T cell activation in mesenteric lymph nodes. Notably, the proportion of CD3^+^CD8^+^ T cells increased by 3.9-fold, confirming that oral administration of this system robustly stimulated mucosal immunity (Fig. 8D).

### 4.3 Nanotechnology based bacterial therapy and CAR-T cell therapy

CAR-T cell therapy represents an autologous immunotherapeutic approach that harnesses a patient's own T cells for personalized cancer treatment. CAR-T cell therapy has shown limited efficacy against solid tumors, primarily attributed to heterogeneous tumor antigen expression, poor T cell persistence, metabolic dysfunction in engineered T cells, and the immunosuppressive TME [94,95]. Moreover, CAR-T cell treatment is frequently complicated by severe adverse events, including CRS (cytokine release syndrome) and ICANS (immune effector cell-associated neurotoxicity syndrome), which arise from excessive immune activation [96]. Nanotechnology offers the advantage of protecting CAR-T cells from the suppressive effects of the TME, while also favorably modulating the pharmacokinetics of immunomodulatory agents through controlled spatiotemporal release. By loading nanomaterials onto and encapsulating CAR-T cells, therapeutic immune modulators can be preferentially trafficked to malignant sites and lymphoid tissues with improved efficiency, thereby promoting CAR-T cell expansion and activity [97,98]. For example, delivering the CAR gene into T cells via nanomaterials can improve gene transfection efficiency while reducing safety concerns associated with viral vectors [99]. Additionally, administering immune checkpoint inhibitors, cytokines, or costimulatory factors through nanomaterial carriers directly to the TME can alleviate tumor-induced immunosuppression and enhance CAR-T cell efficacy [100,101]. Moreover, nanomaterials combined with photothermal or photodynamic therapy can directly ablate tumor cells while releasing TAA, further augmenting the tumor-killing capacity of CAR-T cells [102].

**Fig. 9:**
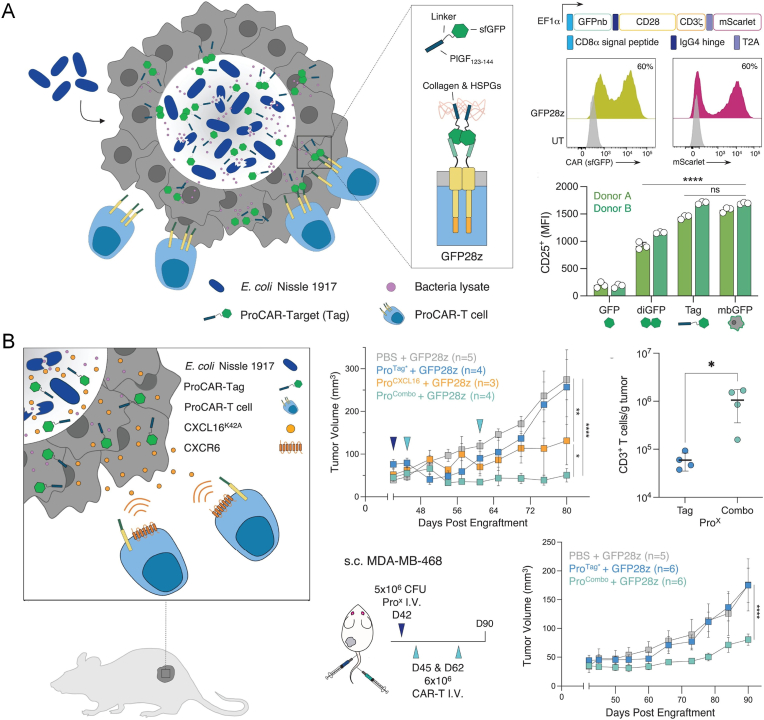
(A) Schematic and flow cytometry analyses demonstrating the ProCAR platform, where tumor-colonizing probiotic bacteria release GFP-based tags to label tumors for GFP-CAR T cell targeting, with validation of CAR expression, reporter coexpression, and T cell activation. (B) Evaluation of mutant CXCL16-expressing probiotics for ProCAR-T cell tumor recruitment, including tumor growth monitoring, TIL quantification, and efficacy assessment in MDA-MB-468 models [104]. Copyright 2023, AAAS.

The application of nanotechnology in CAR-T cell therapy underscores its potential in precision immunotherapy (Fig. 6C). Nevertheless, nano-bacteria hybrids with CAR-T cell therapy remains in the research and exploratory phase. Owing to their ability to selectively colonize the immunoprivileged core of tumors and exert intrinsic immunomodulatory effects, bacteria can be engineered into an antigen-independent platform for targeted immunotherapy. Zhang et al. [103] constructed sialic acid responsive engineering bacteria. *Escherichia coli* MG1655 was designed to harbor a sialic acid-responsive regulatory gene circuit and express hemolysin E (HlyE) through metabolic engineering. By employing bioorthogonal chemistry, the surface of these engineered bacteria was modified with sialidase, enabling the specific recognition and cleavage of sialic acid polysaccharides on tumor cell surfaces, thereby degrading them into free sialic acid. This process mimics the antigen recognition and subsequent cytotoxic response observed in CAR-T cells. Sialidase-mediated desialylation of tumor cells reverses glyco-immune checkpoint-induced immunosuppression, further improving the therapeutic outcome for solid tumors. Bacteria can not only simulate CAR-T therapy through specific metabolic pathways, but also guide CAR-T cell. Danino et al. [104] pioneered a probiotic-guided CAR-T cell (ProCAR) platform where tumor-colonizing bacteria secrete synthetic antigens to achieve spatially restricted CAR-T cell activation. In this innovative system, the researchers first engineered an extracellular "tag" protein (Tag) designed to specifically label the TME. The Tag protein comprises a super-folded green fluorescent protein dimer (diGFP) fused to the heparin-binding domain (HBD) of placental growth factor-2 (PlGF-2), achieving dual functional advantages: (1) The HBD facilitates binding to tumor-associated extracellular matrix components such as collagen, fibronectin, and heparan sulfate proteoglycans (HSPGs), thereby restricting protein diffusion to enhance localization specificity and systemic safety; (2) The diGFP moiety serves as a synthetic antigen that promotes CAR-T cell polarization and amplifies anti-tumor cytotoxicity. To ensure stable Tag expression, the team employed Axe/Txe toxin-antitoxin stabilized plasmids driven by constitutive tac promoters, with in vitro assays confirming robust collagen-binding capacity (Fig. 9A). Immunohistochemical analysis revealed elevated T cell recruitment in tumors treated with the ProCombo regimen. The platform's translational potential was further evidenced by systemic delivery studies: Intravenous administration of engineered probiotics (5 × 10^6^ CFU) achieved 100 % tumor-specific colonization without detectable proliferation in healthy organs. When combined with ProCAR-T cells, this targeted delivery system maintained therapeutic efficacy across multiple human xenograft and murine syngeneic cancer models (Fig. 9B). Mechanistically, the study demonstrated that CAR-T cell activation through probiotic-guided antigen deposition enables antigen-independent bystander lysis while maintaining an exceptional safety profile. These findings establish ProCAR as a modular platform for spatially controlled immunotherapy, addressing critical challenges in CAR-T cell specificity and on-target/off-tumor toxicity.

In summary, the combination of bacterial nanotechnology with CAR-T cell therapy can yield a synergistic antitumor effect by simultaneously modulating the TME and enhancing CAR-T cell functionality. This integrated strategy overcomes the limitations inherent in single-modality treatments—such as tumor heterogeneity, immune suppression, T cell exhaustion, and insufficient infiltration—thereby improving the overall efficacy of precision cancer immunotherapy. Current research integrating bacterial platforms with CAR-T cell strategies remains predominantly limited to adjunctive or combinatorial approaches, rather than establishing truly autonomous “CAR-like” bacterial immune effectors. Although these strategies are conceptually inspired by CAR-T therapy, they have yet to endow bacteria with genuine antigen recognition and cytotoxic capabilities. In theory, it is conceivable to design a bona fide “CAR-bacterium” capable of displaying antigen-binding modules-such as nanobodies-on its surface, which, upon target engagement, would initiate the intracellular signaling cascades required for effector protein release. However, the construction of such a system poses substantial challenges. Its realization hinges on advanced synthetic biology tools, including modular genetic circuits, membrane-associated sensing elements, and tightly regulated promoters, all of which are necessary to achieve highly specific recognition and responsive activation. Moreover, to mitigate risks of systemic toxicity, stringent safety mechanisms must be incorporated, such as suicide switches or auxotrophy-based biocontainment strategies.

## 5 Conclusion and future perspectives

Bacterial therapy has emerged as a promising modality in precision cancer treatment, capitalizing on microorganisms' natural tumor-targeting capabilities, intrinsic immunogenicity, and direct antitumor effects. Over a century ago, clinical observations suggested that bacterial infections could suppress tumor growth. Now, the development of genetically engineered bacteria and nanotechnology-assisted delivery systems has revitalized bacterial therapy as a viable anticancer strategy. Advances in next-generation sequencing and molecular profiling have further deepened our understanding of tumor-resident microbiota and their role in modulating responses to immunotherapy. In particular, the integration of bacterial therapy with established immunotherapies such as ICIs and CAR-T cell therapy has yielded promising synergistic effects, marking a pivotal step toward the clinical translation of bacteria-based immunotherapeutics.

However, standalone bacterial therapy has not yet achieved satisfactory clinical outcomes in cancer treatment, mainly due to its limited cytotoxic efficacy and poor intratumoral penetration. Some animal models have shown that bacterial proliferation may even act as a “accelerator” to tumors, boosting their growth and metastasis. The introduction of large numbers of live bacteria into the body may trigger systemic inflammatory responses and even lead to severe risks such as sepsis [105]. To mitigate these risks, researches have prioritized the use of probiotic strains with higher intrinsic safety like *Escherichia coli* Nissle 1917 and *Bifidobacterium*, or employed genetic engineering approaches to knock out virulence-associated genes, thereby reducing immunogenicity while preserving tumor-targeting capability. In addition, surface engineering of bacteria has emerged as a promising approach to reduce their infection potential in normal organs and minimizing nonspecific colonization, thus achieving higher *in vivo* biosafety [106]. Therapeutic delivery is frequently compromised by an unstable nanomaterial–bacteria interface: weak or labile coupling leads to premature release of cargo prior to tumor accumulation. Feasible countermeasures include the optimization of conjugation methods, such as bioorthogonal covalent coupling and surface engineering approaches to improve nanoparticle or drug attachment and retention on the bacterial surface [107]. Thick or rigid coatings may hinder bacterial chemotaxis and reduce colonization of hypoxic tumor niches, therefore, hierarchically designed systems with an outer protective layer or cleavable linker that is stable in circulation but degrades in response to tumor-specific cues (low pH, proteases, or elevated ROS) are recommended.

In parallel, the development of non-living bacterial derivatives, such as OMVs, BGs, and GVs, offers safer alternatives while preserving therapeutic functionalities. In particular, engineering bacterial OMVs as nanocarriers represents a strategy with high clinical translational potential. Engineered bacteria can directly release immunomodulatory molecules, while engineered OMVs used to deliver immune checkpoint inhibitors can overcome tumor immunosuppression and promote activation of cytotoxic T lymphocytes (CTLs) and natural killer (NK) cells, thereby converting immunologically “cold” tumors into “hot” ones. Moreover, OMV-based nanohybrid systems hold promise to augment CAR-T cell therapy by broadening antigen recognition, improving infiltration into solid tumors, and locally delivering costimulatory signals within the immunosuppressive tumor microenvironment to counteract T cell exhaustion and enhance antitumor efficacy.

Manufacturing processes and quality control represent are fundamental barriers to clinical translation. Live biotherapeutic products cannot undergo conventional terminal heat treatment or filtration methods applied to small-molecule drugs, making it extremely challenging to maintain sterility and batch-to-batch consistency throughout the production process. By contrast, most current cancer therapies still rely on conventional genotoxic modalities such as chemotherapy and radiotherapy, whose dosing and protocols are guided by reliable standards. In the absence of well-established quality benchmarks, it remains difficult to ensure consistent drug-loading efficiency and bacterial viability across different production batches, resulting in challenges for accurate dosing of bacterial therapy. This is also one of the reasons why only a limited number of cell-based therapies have gained clinical approval to date. To address this issue, it is essential to establish a comprehensive GMP framework covering strain preservation, amplification, preparation, purification, and harvesting, accompanied by real-time monitoring as well as standardized seed banks and cryopreservation systems. Moreover, the potential ecological risks associated with live bacterial therapies should not be overlooked, and must be thoroughly assessed prior to clinical implementation.

Recognizing these hurdles, by harnessing the tumor-targeting capacities of bacteria and the tunable physicochemical properties of nanomaterials, nano-bacteria hybrids present a powerful approach for overcoming key limitations of conventional cancer therapies, including inefficient drug delivery and suboptimal immune activation. The combination with other treatment modalities, such as PTT, PDT, chemotherapy, and RT, is expected to address the issues of immune overactivation, cytotoxicity, and production stability. Continued interdisciplinary innovation and translational research will be critical to unlocking their full therapeutic potential and integrating them into standard oncologic care.

## CRediT authorship contribution statement

**Liang Yu:** Writing – original draft, Visualization, Software, Methodology, Investigation, Funding acquisition, Formal analysis, Data curation, Conceptualization. **Yulin Qiu:** Data curation, Formal analysis, Investigation. **Baorui Liu:** Funding acquisition. **Xu Zhen:** Writing – review & editing, Visualization, Project administration, Funding acquisition. **Rutian Li:** Writing – review & editing, Validation, Methodology, Funding acquisition.

## Declaration of competing interest

The authors declare that they have no known competing financial interests or personal relationships that could have appeared to influence the work reported in this paper.

## Data Availability

No data was used for the research described in the article.
